# Rupture of an Infected Renal Cyst Secondary to a Renocolic Fistula in Autosomal Dominant Polycystic Kidney Disease

**DOI:** 10.7759/cureus.4983

**Published:** 2019-06-24

**Authors:** Charlotte Taylor, Miller Jennings, Lakshmi Ramachandran Nair, Manohar Roda

**Affiliations:** 1 Radiology, University of Mississippi Medical Center, Jackson, USA; 2 Pathology, University of Mississippi Medical Center, Jackson, USA

**Keywords:** autosomal dominant polycystic kidney disease, renal cyst infection, renal cyst rupture, renocolic fistula

## Abstract

We present a rare case of retroperitoneal rupture of an infected renal cyst secondary to a renocolic fistula in a patient with autosomal dominant polycystic kidney disease (ADPKD). Intraperitoneal rupture of infected cysts in ADPKD resulting in peritonitis has been described, but to our knowledge, this is the first reported case of retroperitoneal rupture. Cyst infections are a common complication of ADPKD and are difficult to treat, potentially leading to sepsis and death.

## Introduction

Cyst infections are a very serious and relatively common complication affecting patients with ADPKD. Approximately 30%-50% of patients with autosomal dominant polycystic kidney disease (ADPKD) experience some form of renal infection [1-3]. A rare cause of infection of a cyst is a renocolic fistula, which can occur as a complication of colonic diverticular disease [1,4]. Renocolic fistulae may also develop secondarily from inflammatory changes associated with an infected cyst [5]. Rupture of infected renal cysts in ADPKD is a rare complication but has been described previously with those cases describing intraperitoneal rupture with peritonitis [4,6-7]. We present a rare case of retroperitoneal rupture of an infected renal cyst secondary to a renocolic fistula in a patient with autosomal dominant polycystic kidney disease status post renal transplant. 

## Case presentation

A 60-year-old Caucasian man with a history of ADPKD status post renal transplant (on immunosuppression therapy), history of diverticulitis, and hyperparathyroidism status post parathyroidectomy presented to the emergency department with intractable left lower quadrant abdominal pain and occasional chills. He denied fever but did endorse loss of appetite. He was hypotensive and in atrial fibrillation with a rapid ventricular response at presentation with mean arterial pressures in the 70s. On physical examination, his abdomen was soft and tender to palpation in his left lower quadrant. White blood cell count was 10,500 per cubic millimeter (reference range 4,000-10,000), and urinalysis and urine culture were negative for infection. He was admitted to the intensive care unit for septic shock and required vasopressor support.

**Figure 1:**
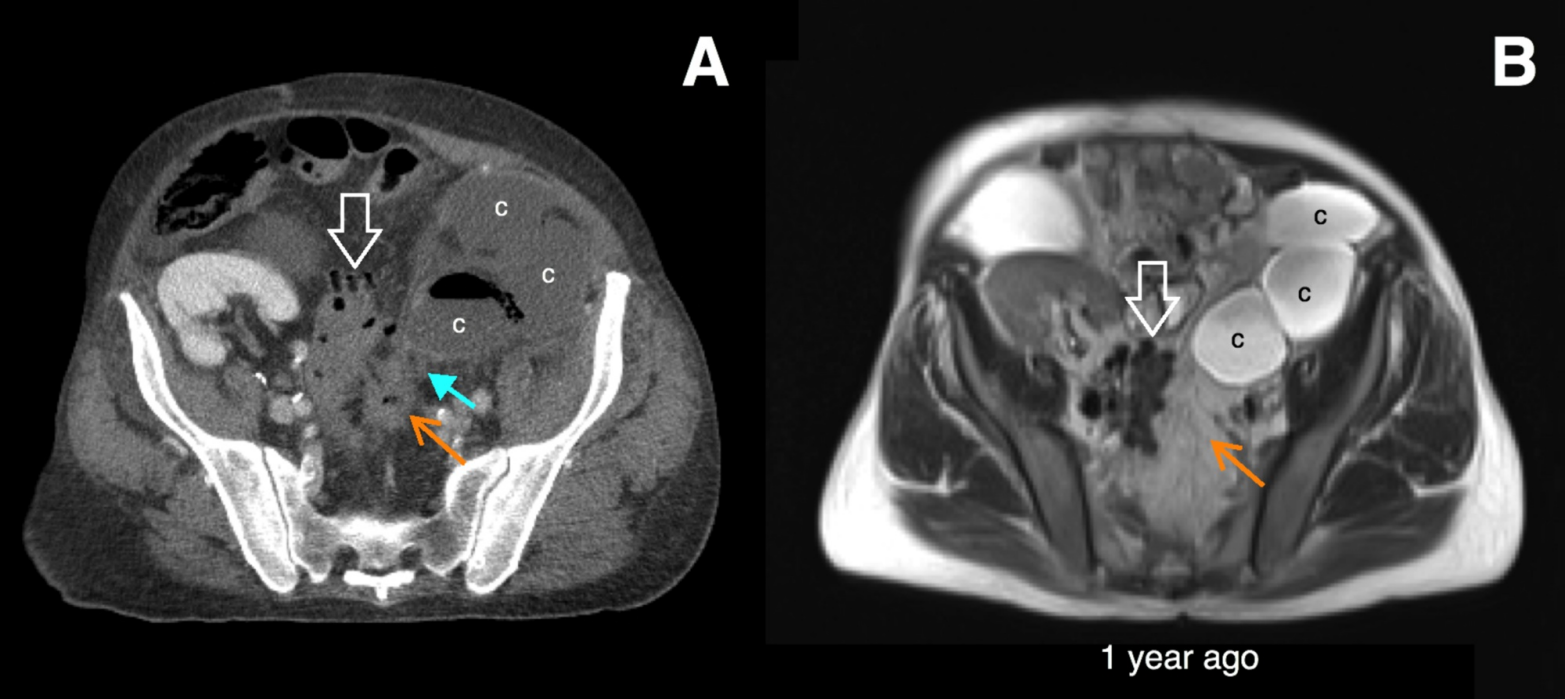
Initial imaging of the cyst infection. Axial computed tomography with intravenous contrast (A) and T2 HASTE magnetic resonance imaging (MRI) (B) show sigmoid diverticulosis (open white arrows) and multiple lower pole renal cysts (c). There is an inflamed diverticulum which is new compared to the prior MRI (orange arrows). Inflammatory stranding is contiguous between the diverticulum and a gas-containing lower pole cyst, which was highly concerning for a renocolic fistula. Right lower quadrant transplant kidney is also seen.

Computed tomography of the chest, abdomen, and pelvis (CT CAP) with intravenous contrast showed markedly enlarged polycystic native kidneys consistent with ADPKD with air in a few cysts in the lower pole of the left kidney concerning for an infected cyst/emphysematous pyelonephritis. Extensive sigmoid colonic diverticulosis was also present, and there was new inflammatory stranding of a sigmoid diverticulum which was contiguous with the lower pole of the left kidney, concerning for a possible renocolic fistula from acute vs. chronic diverticulitis (Figure 1). The patient was treated for a cyst infection with broad-spectrum intravenous antibiotics (piperacillin-tazobactam, vancomycin, and levofloxacin).

**Figure 2:**
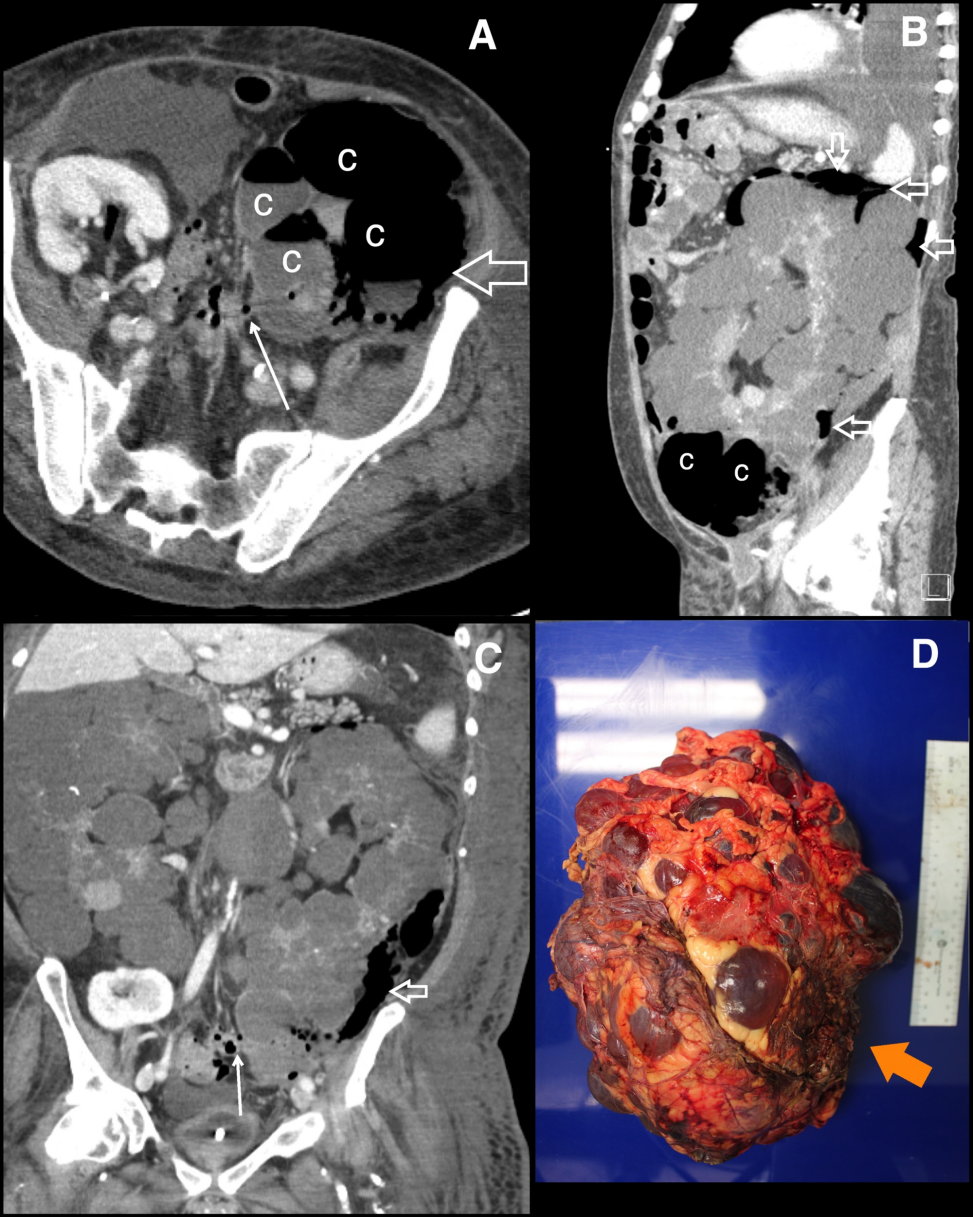
Retroperitoneal rupture of infected renal cysts. Axial (A), sagittal (B), and coronal (C) computed tomography of the chest, abdomen, and pelvis post-contrast images nine days after initial presentation reveal more numerous air and fluid-filled cysts (c) with gas tracking from the cysts into the perirenal and retroperitoneal spaces (open white arrows). This is compatible with rupture with retroperitoneal abscess formation. The renocolic fistula is better seen on this study with air tracking along the sinus tract from the sigmoid colon to the lower pole cysts (small white arrows, A and C). Nephrectomy specimen (D) shows a markedly enlarged kidney with innumerable intact cysts with little intervening renal parenchyma and ruptured lower pole cysts (orange arrow).

Nine days later, the patient had been stabilized and transferred to the floor, but he had worsening leukocytosis (WBC count now 32,600/cm^3^). Repeat CT CAP (Figure 2) showed increased gas within multiple lower pole cysts in the left kidney with a large perirenal gas containing fluid collection concerning for ruptured infected renal cyst with perirenal abscess formation. The patient was taken to the operating room for a left radical nephrectomy and retroperitoneal washout. Intraoperative findings and subsequent pathology findings confirmed the presence of ruptured infected lower pole cysts and a perirenal abscess (Figure 2). The causative organism was not isolated from intraoperative cultures.

Postoperatively, his course was complicated by multiple intra-abdominal abscesses requiring drainage by interventional radiology, *Bacteroides fragilis* septicemia, and acute kidney injury. His acute kidney injury and leukocytosis improved, and he was discharged to a skilled nursing facility six weeks after presentation.

## Discussion

Renal cyst infections have different etiologies. Retrograde infection from the lower urinary tract is suspected to be the most common cause of upper urinary tract infections in patients with ADPKD, given the female predominance and the presence of enteric organisms [6]. A fistulous connection to the bowel (renocolic fistula), which can occur secondary to trauma or adjacent colonic infection/inflammation, is a rare cause of cyst infection [8-9]. Our patient had an episode of diverticulitis resulting in the formation of a fistulous tract to the lower pole cysts in his left kidney, allowing bacteria from the colon to enter the cyst. This resulted in a fulminant infection presenting clinically as septic shock. Our patient was particularly susceptible to infection given his active immunosuppression for his kidney transplant.

An even more rare cyst complication, rupture of infected renal cysts in ADPKD has been described previously in the literature with those cases describing intraperitoneal rupture with peritonitis [4,6-7]. In the case of our patient, the rupture was perirenal/retroperitoneal, which to our knowledge has not been previously reported in the literature. Mabillard et al. described retroperitoneal rupture of hemorrhagic cysts in ADPKD leading to retroperitoneal hematoma [10]. The rupture of infected cysts, whether intraperitoneal or retroperitoneal, is pathophysiologically the same. Infection and increased pressure resulting in cyst wall necrosis and rupture. The clinical presentation, however, may be different. Intraperitoneal cyst rupture causes peritonitis and will present clinically with severe tenderness, distention, and guarding [6]. Retroperitoneal rupture may present as flank pain.

Imaging of the cyst complication in ADPKD was relatively straightforward in our patient. The presence of gas in a cyst on CT is highly concerning for infection, either from gas-forming organisms or from a fistula with bowel. In the absence of gas, the diagnosis of a cyst infection is more difficult. The cyst may be thick-walled and contain complex fluid. Complex fluid in a cyst on ultrasound manifests as internal echogenic debris and does not follow simple fluid signal on magnetic resonance imaging. Diffusion restriction may also be present in infected cysts. However, the presence of complex internal fluid does not equate to infection, and the clinical presentation should be taken into account (sepsis, abnormal urinalysis, positive urine cultures). Also, the thick cyst wall may be nodular and demonstrate enhancement, raising concern for renal cell carcinoma, for which these patients are at increased risk [11]. The diagnosis of renocolic fistula may be confirmed with enteric contrast, either ingested orally or via barium enema. The presence of enteric contrast within the cyst is diagnostic. Enteric contrast was not given in our case; however, the fistula was clearly visible with air along a sinus tract from the sigmoid diverticulum to the gas-containing cyst.

Infections involving cysts are particularly difficult to treat, as antibiotic penetration of cysts is generally poor. Lipid soluble intravenous antibiotics show the best cyst penetration [2]. Infections secondary to renocolic fistulae may be refractory to antibiotic therapy alone.

## Conclusions

Infected renal cysts should be in the differential diagnosis of any patient with ADPKD with leukocytosis and/or fever. Diagnosis of a renal cyst infection by imaging may be challenging due to the potential complexity of the cysts in ADPKD. The etiology of the infection should be sought, as management is different if a fistula is present, necessitating a nephrectomy rather than intravenous antibiotics alone.
